# Whose mu fared better? The effect of extended-release naltrexone (XR-NTX) on treatment outcomes for opioid and alcohol users

**DOI:** 10.1186/1940-0640-10-S1-A9

**Published:** 2015-02-20

**Authors:** Desirée A Crèvecoeur-MacPhail

**Affiliations:** 1 Semel Institute for Neuroscience and Human Behavior, David Geffen School of Medicine, University of California, Los Angeles, CA, 90025, USA

## Background

In 2010, Los Angeles County made the decision to offer publicly funded patients receiving treatment for substance use disorders extended-release naltrexone (XR-NTX, brand name “Vivitrol”). Since 2010, over 1200 patients have received at least one dose of extended-release naltrexone. Little is known concerning the differential impact of the medication on those who use it for alcohol use disorders (AUDs) when compared to patients who take the medication for opioid use disorders (OUDs).

## Materials and methods

Quantitative data were collected on total doses received and treatment characteristics for patients who received XR-NTX as well as evaluation data such as urges to drink alcohol or use opioids, reported side effects, and substance use (n = 451). Data were collected at baseline, once a week for the first 3 weeks after the initial dose, and once a month thereafter, as long as the patient took the medication. In addition, data were collected for a subset of 114 individuals at 30 and 60 days after the final dose administration.

## Results

XR-NTX was associated with good engagement and retention rates. Both groups of patients, those with AUDs and those with OUDs, reported side effects, but opioid users reported more side effects in the first week. On average, patients in treatment for OUD took slightly fewer doses when compared to patients with AUDs; however, this difference was not statistically significant (p = 0.66). Both groups (OUD and AUD) demonstrated reductions in urge to drink (p < 0.001) and urge to use opioids (p < 0.001), and these reductions were maintained at the two follow-up points.

## Conclusions

The use of XR-NTX for patients with AUDs and OUDs is associated with good engagement and retention rates in treatment programs. Side effects were reported but minimal, and although there were some differences in the number of doses taken by primary drug (opioids or alcohol), both groups reported sustained reductions in urges and substance use at the follow-up.

